# Separating Amplitude Response from Apparent Cyclic Drift in Self-Sensing Cementitious Composites

**DOI:** 10.3390/s26196353

**Published:** 2026-10-08

**Authors:** Shaban Shahzad, Waqas Rafiq, Mohammed A. Al-Osta, Asad Hanif, Salman Siddique

**Affiliations:** 1 Interdisciplinary Research Centre for Construction and Building Materials, King Fahd University of Petroleum and Minerals (KFUPM), Dhahran 31261, Saudi Arabia; shaban.shahzad@kfupm.edu.sa (S.S.); malosta@kfupm.edu.sa (M.A.A.-O.); asad.hanif@kfupm.edu.sa (A.H.); 2 Department of Civil Engineering, Faculty of Engineering, University of Tabuk, Tabuk 71491, Saudi Arabia; waqas.rafiq@ut.edu.sa; 3 Civil and Environmental Engineering Department, King Fahd University of Petroleum and Minerals (KFUPM), Dhahran 31261, Saudi Arabia

**Keywords:** self-sensing concrete, carbon nanotubes, piezoresistivity, gauge factor, structural health monitoring, experimental design

## Abstract

The loading sensitivity of a self-sensing cementitious composite depends on the stress amplitude at which it is measured. That dependence is established, but its consequence for the protocol used to obtain the sensitivity has not been examined. Conventional protocols apply a few cycles at ascending amplitude levels, so the between-level component of cycle number is fixed by amplitude. The present study treats this as an identification problem: only the within-level repeated information can inform a cycle trend, and the data cannot identify a general cycle-history effect independently of amplitude. Three multi-walled carbon nanotube concrete cylinders of identical composition, one for each conditioning history, were investigated using a four-probe alternating-current method at 1 kHz under a three-level stepped-amplitude protocol in two loading variants, then loaded monotonically to failure. Across the six cyclic records, the correlation between cycle number and peak stress is r = 0.87 to 0.90, and 60% to 92% of the variance in loading sensitivity lies between amplitude levels rather than within them. With amplitude held fixed, within-level slopes are small and inconsistent in sign; however, three cycles per level cannot establish that a cycle effect is absent. Sensitivity magnitude falls between the middle and highest amplitude blocks in all six records, at 2.3 to 11.2 standard errors of the difference, three of which exceed their comparison-specific Welch critical value. At the lower amplitude step, the largest resolved difference is 0.673%/MPa in the continuous record of C1, whereas the partially saturated C2 records show no resolvable difference. Because amplitude and block order advance together under this protocol, these are differences among amplitude blocks and are not attributed to amplitude in isolation. The moisture-related pattern is an association across three individual specimens, not a replicated moisture effect. Monotonic loading provides a within-specimen extrapolation demonstration under the selected response family, not independent predictive validation. An experimental design with fixed-amplitude replication and a nominal precision calculation is specified.

## 1. Introduction

Deterioration of concrete structures begins with cracking, and detecting that cracking before it becomes structurally consequential has motivated a large body of monitoring research [1,2,3]. Conventional approaches attach discrete instruments to the structure: acoustic emission transducers [4], plastic or distributed optical fibres [5,6,7], and embedded piezoelectric elements [8]. Each requires a sensing element distinct from the structure, with the associated durability, coverage and installation limitations.

An alternative is to make the material itself the sensing element. Cementitious composites containing conductive fillers respond to mechanical loading with a measurable change in electrical resistance, and this property has been developed over three decades as a route to self-sensing structures [9,10,11,12,13,14]. Carbon fibres were the first filler used at scale [13,14,15], and their behavior as a sensing phase in cementitious matrices has been studied since the early 1990s [16,17,18]. The discovery of carbon nanotubes [19] and the subsequent characterization of their electrical and mechanical properties [20,21,22,23] introduced a filler of far higher aspect ratio, which percolates at lower dosage [24] and has since been applied widely in cementitious systems [25,26,27]. Reviews of these piezoresistive materials and of their measurement practice are available [10,28,29,30], and comparative studies of nanocarbon fillers report gauge factors spanning two orders of magnitude between systems [31].

The transduction is commonly described by a gauge factor (GF), GF = (ΔR/R_0_)/ε, or by loading sensitivity S = d(FCR)/dσ. GF is strain-normalized, whereas S in this study is the ordinary least-squares slope of FCR against stress over a loading branch. They are not interchangeable, and GF is not calculated from the present records. Both quantities are nevertheless often reported as single figures characterizing a mix, and comparisons between mixes, fillers and hybrid systems are made on that basis [32,33,34,35,36,37,38].

It is already established that the coefficient is not a material constant. Chung reports a stress sensitivity falling from about 1.7 × 10^−5^ to 0.4 × 10^−5^ Pa^−1^ between low and higher loads [39]. Roshan et al. [40] used 4% MWCNT/GNP in cement-stabilized sand and reported stress sensitivity increasing with stress level. These values are not directly comparable to the present 0.75% MWCNT concrete because the matrix, filler dosage, electrode configuration, and normalization differ; they nevertheless show that response direction and magnitude are loading dependent. The non-linearity of the relationship between fractional change in resistance and strain has been noted, fitted with polynomial forms, and modelled explicitly with a Ramberg–Osgood formulation on the argument that ignoring the non-linear portion limits the monitoring range [41]. Comparisons of two-probe and four-probe measurement configurations are framed in terms of how much the gauge factor varies with strain amplitude [42]. A non-linear response curve is an amplitude-dependent coefficient by definition, and this is reported in the literature. Reviews have begun to identify the collection and analysis of characterization data as a methodological limitation in this literature [43].

Here, cyclic drift denotes an apparent systematic change in a sensitivity estimate with successive cycle number when nominal loading conditions are treated as comparable. It is not synonymous with general loading-history effects, which can include dwell, unloading and recovery, damage, temperature, and moisture evolution. Conventional characterization applies a few load cycles at each of several ascending amplitude levels, three cycles at each of three levels being a common arrangement [44,45,46]. Under such a programme, amplitude is constant within a block but advances with the cycle block. An across-cycle trend is therefore compatible with an amplitude response, and the present study asks whether the two effects are independently identified rather than merely whether they are correlated. Three cycles at each level leave only three repeated observations per level for examining a cycle trend.

A mechanistic reason may exist for the dependence, but the mechanistic interpretation in this study is a hypothesis rather than an experimentally demonstrated decomposition. Conduction can involve ionic transport through the pore solution and electron transfer between filler particles, and models of piezoresistivity in these materials have been built on that basis [47,48,49]. Polarization effects and impedance measurements can distinguish contributions under suitable conditions [27,50,51], while the balance between them may shift with the moisture state of the specimen [52]. The present measurements do not include pore–solution conductivity, impedance decomposition, or inter-particle distance measurements. Load can alter the two pathways differently, and the resulting amplitude dependence need not have the same sign in every material. The reported direction is indeed not universal: an increase in stress sensitivity with stress level has been reported for cement-stabilized sand containing 4% MWCNT/GNP [40], whereas the present records generally show a decrease.

This study examines the identification problem and quantifies it. Section 4 quantifies the differences among amplitude blocks in the present material, level by level and with an explicit within-record uncertainty treatment. Section 5 provides the factor representation, separates between-level from within-level information, and shows why a flexible cycle-history effect is not identifiable under the adopted protocol. Section An Experimental Design That Separates the Two Effects specifies a replicated fixed-amplitude design and gives a nominal precision calculation for the proposed cycle count. Section 6 evaluates, on the same specimens, a stress-ratio-denominated within-specimen extrapolation and states why its held-out errors are not independent predictive validation. All three specimens and the underlying electrical and mechanical records belong to the previously reported programme [45,46]. The fabrication, conditioning, loading, and recording were inherited from that programme; the present study introduces the reanalysis of those records by resistance referencing, peak detection, amplitude clustering, per-level uncertainty analysis, factor identifiability analysis, and monotonic truncation tests. The figures and tables in Section 4, Section 5 and Section 6 are regenerated from this analysis, and no new experimental measurements were collected for this study.

## 2. Materials and Experimental Programme

### 2.1. Materials and Mix Proportions

All specimens came from a single concrete batch based on ordinary Portland cement CEM I 52.5R conforming to EN 197-1:2011 [53], with natural sand of 0 to 4 mm and natural coarse aggregate of 4 to 10 mm. Multi-walled carbon nanotubes supplied by Nanocyl (Sambreville, Belgium) were added at 0.75% by mass of cement. The previously reported characterization of this material lists an average diameter of 9.5 nm, an average length of 1.5 μm, carbon purity of 90%, a specific surface area of 250–300 m^2^/g, and a powder volume resistivity of 10^−4^ Ω·cm [54,55]. The product grade was not retained in the source programme and has not been inferred here. Dosage and dispersion route follow the development reported previously for this composite [54,55]. Mix proportions are given in Table 1.

Dispersion method governs the electrical and mechanical properties obtained from a given nanotube dosage [26,54,55], so the available procedure is described in full. The nanotubes were combined with the mixing water and stirred mechanically, then the suspension was sonicated with a UP400St ultrasonicator (400 W, 24 kHz; Hielscher Ultrasound Technology, Teltow, Germany) at one minute per litre until uniformly black. The nominal equipment energy input was therefore 24 kJ/L at the rated power, although delivered acoustic energy and suspension temperature were not measured. Dispersion was verified visually by the uniformly black appearance; no microscopy, spectroscopy, or batch-level homogeneity measurement was retained. Cement, sand and coarse aggregate were dry-mixed; the dispersed suspension was then added gradually during mixing, followed by the superplasticiser. Specimens were demoulded after 24 h and cured for 28 days at 20 °C and 100% relative humidity.

### 2.2. Specimens and Electrode Configuration

**Figure 1:**
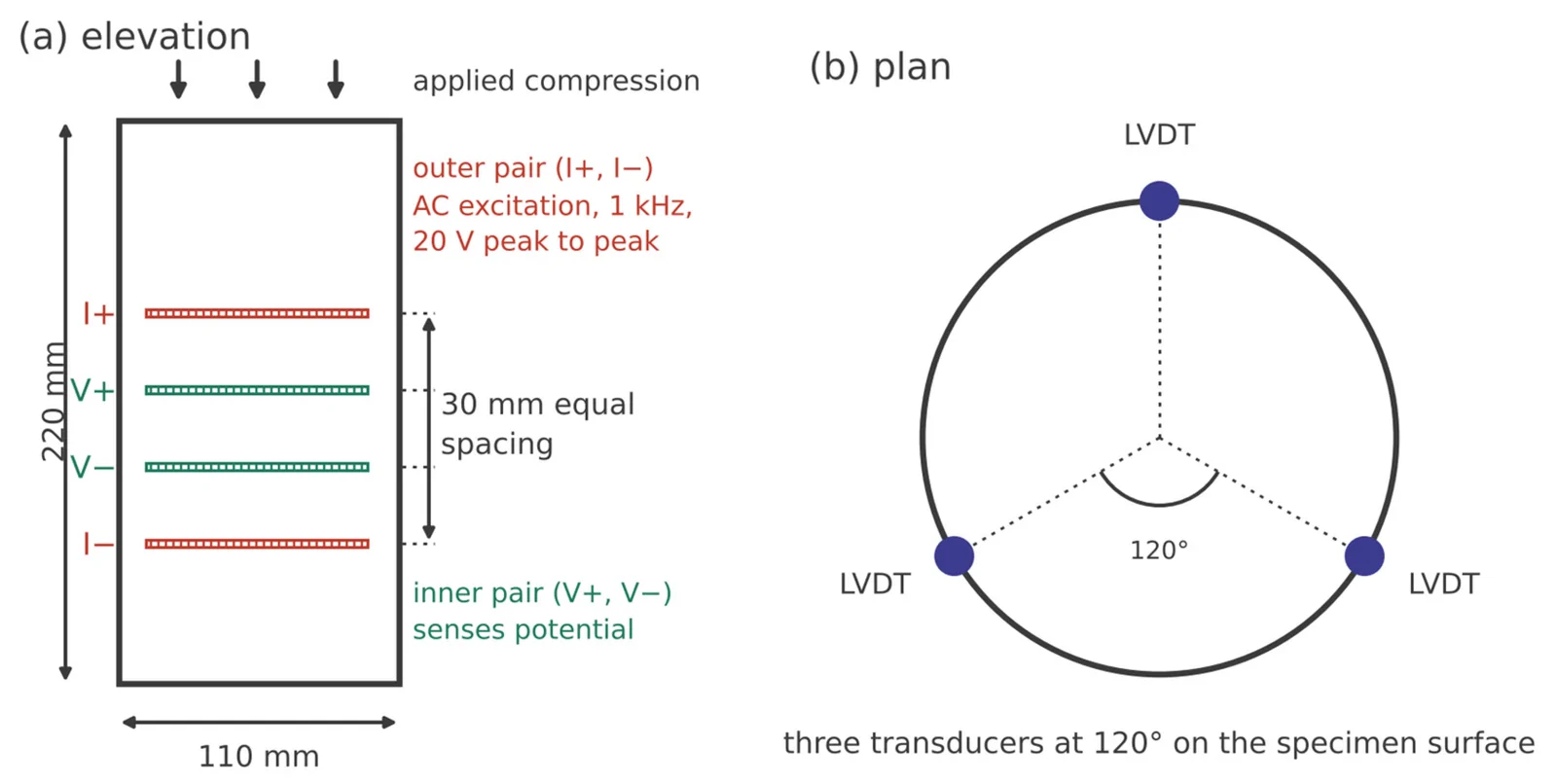
Specimen and electrode layout. (**a**) Cylinder, 110 mm across and 220 mm high, with four gauze electrodes at 30 mm spacing. The outer pair drives the 1 kHz excitation, the inner pair reads the potential. (**b**) Three LVDTs at 120°.

Concrete cylinders of 110 mm diameter and 220 mm height were cast with four stainless steel gauze electrodes positioned in the mould before pouring, centred on the specimen axis, and spaced 30 mm apart. The outer pair carried the excitation current and the inner pair sensed the resulting potential, a four-probe arrangement that excludes contact resistance at the current-carrying electrodes from the measured quantity. Excitation was an alternating voltage of 20 V peak-to-peak at 1 kHz. In this reanalysis, resistance denotes the scalar resistance channel supplied by the four-probe acquisition system, interpreted as the ratio of the potential measured by the inner pair to the current through the outer pair. Phase-resolved impedance components were not retained, so this channel is not interpreted as a complex impedance magnitude. The source programme does not preserve the sampling frequency, averaging window, phase settings, or instrument uncertainty, and these parameters cannot be reconstructed retrospectively. Axial strain was measured by three linear variable differential transducers spaced at 120° around the specimen. The configuration is shown in Figure 1.

### 2.3. Conditioning States

After curing, the three specimens were conditioned to three distinct environmental histories, designated C1, C2, and C3 (Table 2). The reported water-mass loss was calculated as L_m_ = 100(m_i_ − m_f_)/m_i_, where m_i_ and m_f_ denote the specimen masses before and after conditioning, respectively. The available source records retain only the percentage values and nominal environmental ranges; conditioning duration, individual initial and final masses, balance precision, time between conditioning and electrical testing, and time-resolved temperature and relative-humidity histories were not preserved. A constant-mass equilibrium therefore cannot be verified. C1, C2, and C3 are operational labels for conditioning histories: C1 is associated with 0% recorded mass loss, C2 with 2.2%, and C3 with 5.1%; degree of saturation was not measured directly. The testing temperature was not separately recorded and is not treated as a controlled factor in comparisons among the three records.

### 2.4. Loading Protocols

Each specimen was subjected to a cyclic compressive programme of three cycles at each of three nominal stress levels, at 20%, 35%, and 50% of the compressive strength, giving nine loading branches. Peak stresses realized in the three levels were approximately 8.8, 17.5, and 23.7 MPa, corresponding to 0.17 to 0.52 of each specimen’s own monotonic peak. Two variants of the cyclic programme were recorded for every specimen. In the continuous variant, the load cycled without pause. In the held variant, a sustained hold of nominally 20 s was applied at the extremes of each cycle. The hold was terminated manually rather than automatically, so the achieved dwell varies between specimens; median dwells were 18.6, 11.9, and 18.0 s for C1, C2, and C3, respectively. The hold occurs at the trough as well as at the peak. The held variant is therefore a hold-at-both-extremes protocol and not a peak-held one, and any contrast between the two variants must be attributed on that basis.

Following the cyclic programme, each specimen was loaded monotonically to failure with the electrical response recorded continuously. The strengths configured in the testing machine, implied by the stress-ratio channel, were 47.9, 46.2, and 52.1 MPa for C1, C2, and C3. The source record does not identify whether the continuous or held cyclic variant was performed first. Because the second test could inherit residual mechanical or electrical history from the first, comparisons between variants are descriptive and are not interpreted as causal protocol effects.

## 3. Measurement Model and Data Handling

### 3.1. Referencing Convention

Fractional change in resistance (FCR) is defined as FCR = 100(R − R_0_)/R_0_, where R is the scalar resistance channel and R_0_ is the reference resistance. The reference used throughout is the first resistance sample of each recording. The recordings contain no preload segment, so load onset was located independently under four detection rules: a strict-increase criterion, a fixed absolute rise, a fraction of the loading span, and a sustained-derivative criterion. Under the sustained-derivative rule, onset falls at the first recorded sample in all six files. At most three leading samples lie within a tight band of the first sample in any file, and none lie within it in three of the six.

The consequence of the reference choice was quantified rather than assumed. Referencing instead to the latest plausible onset shifts the reference by 0.04% to 0.97%; because FCR is defined relative to that reference, every fitted sensitivity, level difference, and associated standard error in a record scales by the same positive factor, while the coefficient of determination and all standardized differences are unchanged. The absolute sensitivity values can therefore move by less than 1%, but the sign, within-record resolved or unresolved classification, and the reported cross-level pattern are not altered. The reference is a scale convention with a known multiplicative effect rather than a physical claim, and a standardized reference-loading stage should be used in future experiments.

Each source file also carries a zeroed resistance channel that is re-referenced part way through the recording, and a raw FCR channel referenced to an externally measured baseline taken during unloading. Neither channel is used in any analysis reported here. In one specimen, the external reference channel cannot be expressed as a single-reference FCR of its own resistance channel at all, disagreeing with that channel by about ten percentage points at failure. That inconsistency is confined to a channel not used in any of the analyses presented here, and is noted so that readers consulting the raw data do not misinterpret it.

### 3.2. Cycle Detection

Loading branches were located by peak detection on the stress channel, using the prominence criterion implemented in the analysis tool together with a height floor, both expressed as fractions of the stress range of the file. A peak was retained when it passed the prominence test and its height was at least 0.30 of the stress range. The numerical prominence setting was not preserved with the source files, so a complete numeric peak-detection specification cannot be reconstructed retrospectively. The height floor determines what is analyzed and is therefore stated explicitly. Any floor above 0.6 of the stress range sits above the lowest of the three designed amplitude levels and excludes it without explicit indication, leaving six of the nine designed cycles and only two of the three amplitude levels. A floor of 0.30 recovers all nine peaks in three well-separated levels on all six records and introduces no spurious peaks; it is used for every result reported here. The analysis also reports the number of prominence-passing peaks falling below the floor, so that an excluded amplitude level is reported to the analyst rather than passing undetected. The 0.30 value was selected from this recovery and false-peak check, not from an independent calibration dataset.

## 4. Variation in Loading Sensitivity Among Amplitude Blocks

### 4.1. Method

For each loading branch, loading sensitivity S was taken as the ordinary least-squares slope S = Cov(σ, FCR)/Var(σ) of FCR against stress σ over the loading half of the cycle, retaining its sign. Branches were then grouped into amplitude levels by clustering on peak stress, a new level beginning wherever the relative gap between successive sorted peaks exceeds a declared tolerance of 0.15. Within-level peak spread in these records is 3% to 6% and between-level spread is about 30%, so the level assignment is unambiguous. Grouping was performed independently of cycle order and recovered the designed structure of three cycles per level in all six records. 

Each level is summarized by its mean peak stress, its mean sensitivity and the within-level standard deviation over three cycles (Table 3). For adjacent levels ℓ and ℓ + 1, the reported difference is Dℓ,ℓ + 1 = S^−^ℓ + 1 − S^−^ℓ and its standard error is SE(Dℓ,ℓ + 1) = [sℓ^2^/nℓ + sℓ + 1^2^/nℓ + 1]^1/2^, with nℓ = nℓ + 1 = 3. The three branch estimates are treated as repeated within-specimen observations for estimating scatter; they are not independent specimen replicates. Because the source analysis has only three cycles per level and retains rounded level summaries rather than all branch-level values, confidence intervals for the within-level slopes cannot be reconstructed reliably. The adjacent-level ratios in Table 4 are therefore used as a within-record precision check, not as between-specimen hypothesis tests. Each difference is analyzed against its own threshold rather than against a common threshold. Welch–Satterthwaite degrees of freedom are evaluated for every comparison as ν = (sℓ^2^/nℓ + sℓ + 1^2^/nℓ + 1)^2^/[(sℓ^2^/nℓ)^2^/(nℓ − 1) + (sℓ + 1^2^/nℓ + 1)^2^/(nℓ + 1 − 1)], and the two-sided 5% critical value t_0.975_,ν is taken at that ν. Across these records, ν ranges from 2.0 to 4.0 and the corresponding critical value from 2.78 to 4.27, so a single fixed 3.2-SE threshold would be permissive for some comparisons and conservative for others. A difference is reported as resolved only where its SE ratio exceeds its own critical value, and both quantities are listed for every comparison in Table 4.

Fitted responses across amplitude are reported only because three distinct levels are available. With two levels a regression of sensitivity on peak stress has only two distinct abscissae, so its slope is fixed entirely by the two group means: it cannot distinguish a linear response from a step, cannot reveal curvature, and has no meaningful residual structure. Three levels are the minimum at which the shape of the response can be examined at all.

### 4.2. Results

**Figure 2:**
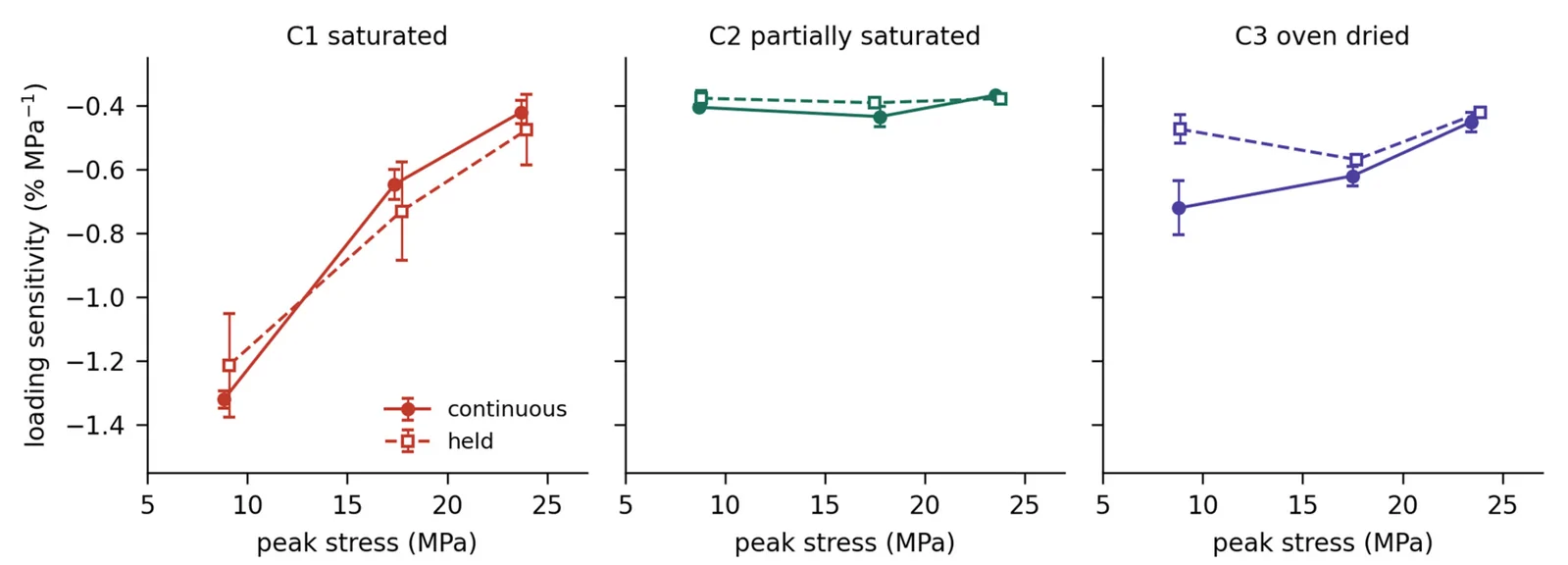
Loading sensitivity against peak stress in the three conditioning histories. Filled circles joined by solid lines, continuous cycling; open squares joined by dashed lines, cycling with holds. Each panel is drawn in a single color that identifies its conditioning history; color carries no other meaning. Bars are the standard deviation over three cycles. All panels share the *y*-axis, and sensitivity keeps its sign, so values nearer zero indicate a weaker response.

Per-level summaries and fitted responses are given in Table 3 and shown in Figure 2. Differences among the amplitude blocks are present in every record. Because block order and amplitude advance together under this protocol, these are reported as differences among amplitude blocks and are not attributed to amplitude alone. Their comparison across C1, C2, and C3 is an association across three individual specimens rather than a replicated moisture effect. C1 has the largest sensitivities at the lowest amplitude, roughly three times those of the other records, and declines steeply and monotonically in magnitude across successive amplitude blocks under both protocols, with coefficients of determination of 0.956 and 0.855. C3 declines monotonically under continuous cycling. C2 shows weak and non-monotone block-to-block differences, with fitted coefficients of determination 0.154 and 0.005. The coefficients of determination describe fits to three level means within individual records; they do not establish a general moisture-dependent material relationship.

The near-zero fitted slopes for C2 and for the held C3 record should not be read as evidence of no effect. They are linear fits that describe responses poorly when those responses are not monotone across the full range, and for those records the per-level summaries and the level differences provide the more appropriate description.

### 4.3. Which Changes Are Resolved

**Figure 3:**
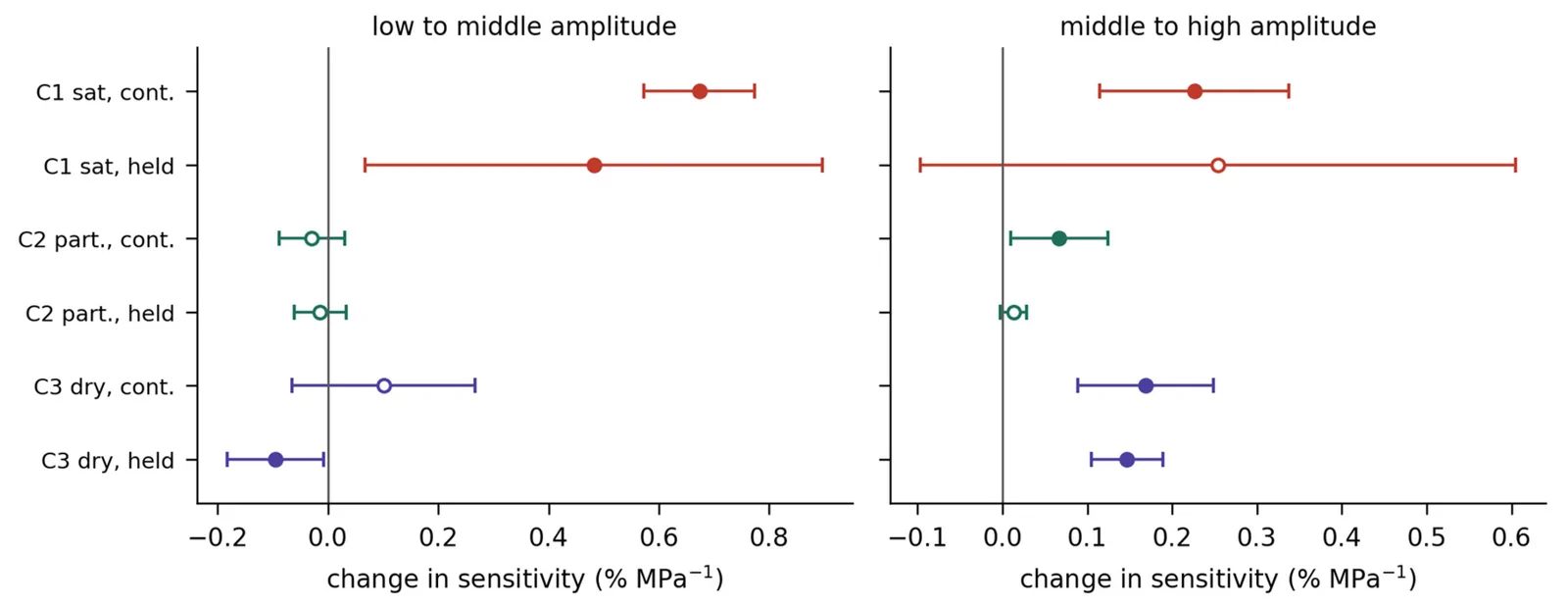
Differences between adjacent amplitude levels, in %/MPa, with bars drawn at the comparison-specific Welch critical value of each difference, as listed in Table 4. Filled markers exceed their own critical value, open markers do not. Colors identify the conditioning history, as labeled on the left axis; the vertical gray line marks zero. A positive difference indicates that the response weakens as amplitude increases.

Table 4 gives every adjacent level difference with its standard error, and the same differences are plotted in Figure 3. The results separate into a resolved within-record component and an unresolved one. Between the middle and the highest amplitude block, sensitivity magnitude falls in all six records, with standardized differences of 2.3 to 11.2; three of these exceed their comparison-specific Welch critical value. Between the lowest and the middle block, two of the six record-level differences exceed their own critical value. These are resolved contrasts within the tested records, not evidence of a reproducible effect across specimens or conditioning states.

Between the lowest and the middle amplitude block, the outcome is not uniformly resolved. The two saturated records exceed their critical values positively, at 21.5 against 3.11 and at 3.7 against 2.78. The held oven-dried difference, at 3.5 standard errors against a critical value of 3.52, lies on the boundary and is not counted as resolved. The remaining three differences, comprising both partially saturated records and the continuous oven-dried record, lie between 1.0 and 1.9 standard errors against critical values of 3.54 to 3.94 and are indistinguishable from the within-record scatter. The shape of the response at the low-amplitude end is therefore resolved for two records and undetermined for four.

That distinction matters for how the continuous oven-dried record is described. Its coefficient of determination of 0.809 and its monotone appearance rest almost entirely on the upper step, resolved at 6.8 standard errors against a critical value of 2.78; its lower step, at 1.9 standard errors against a critical value of 3.54, is not resolved. Reporting the fit alone would imply that the entire response is established, which it is not. Fitted responses are therefore reported here alongside the level differences rather than in place of them.

## 5. Confounding of Amplitude with Cycle Number

**Figure 4:**
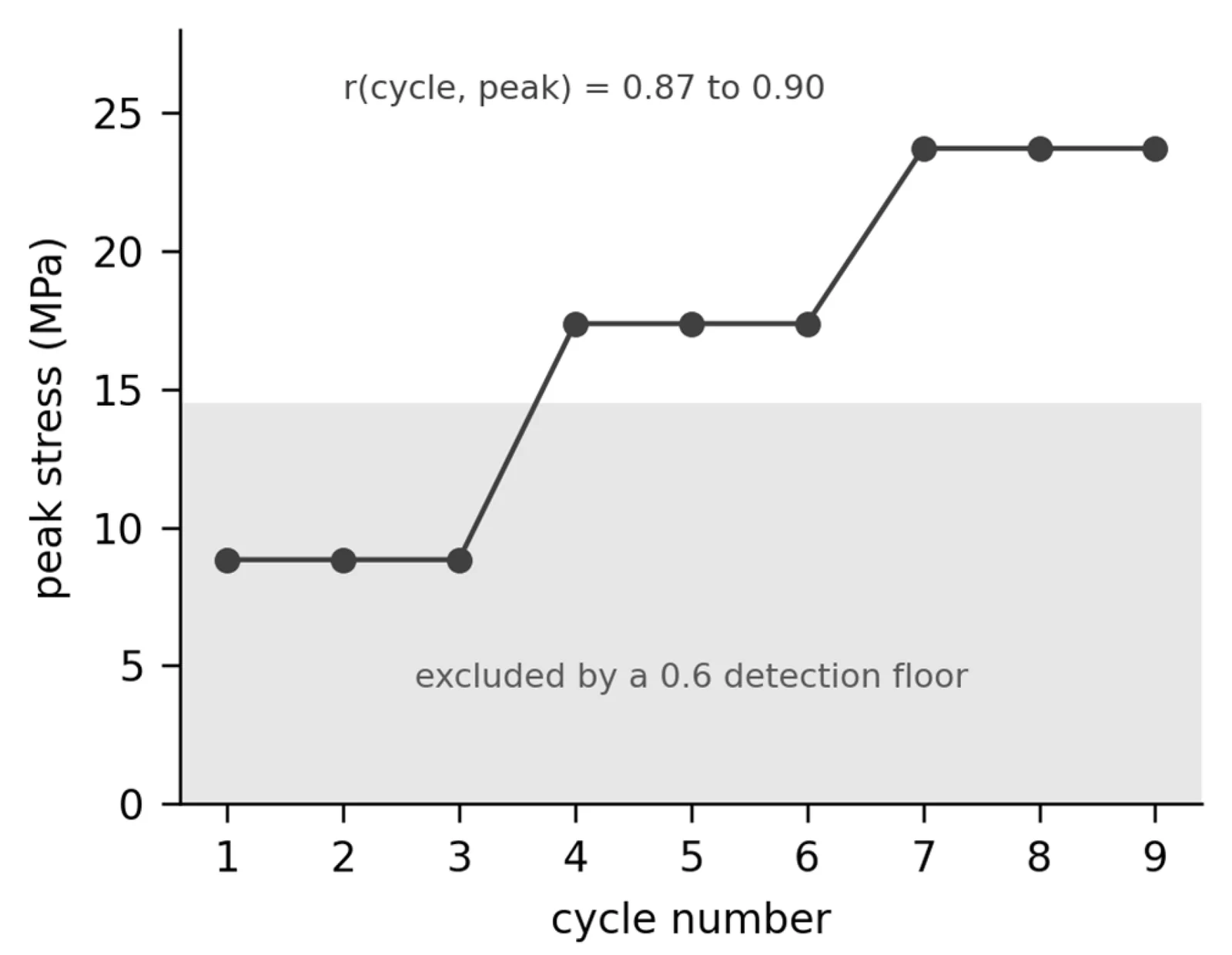
Peak stress increases with cycle number by design, so the two correlate at r = 0.87 to 0.90 and 60% to 92% of the variance in sensitivity lies between levels. Shading indicates the data that a detection floor at 0.6 of the stress range would exclude.

The stepped-amplitude protocol applies a small number of cycles at each of several ascending amplitude levels, so cycle number and peak stress are correlated by construction. In these records the Pearson correlation between them is r = 0.87 to 0.90 across the six records. The factor structure can be written for a nine-branch record as c = (1, 2, 3, 4, 5, 6, 7, 8, 9), ℓ(c) = (1, 1, 1, 2, 2, 2, 3, 3, 3), and w = c − c^−^ℓ = (−1, 0, 1, −1, 0, 1, −1, 0, 1), where c^−^ℓ = (2, 5, 8) is the cycle-block mean. A general additive identification model is Y_s,p_,ℓ,k = α + f(Aℓ) + g(c) + ε, where Y is loading sensitivity, s is specimen, p is protocol, Aℓ is amplitude level, and k is the within-level cycle. Since c = c^−^ℓ + w and c^−^ℓ is in one-to-one correspondence with Aℓ, the between-block part of g(c) is aliased with f(Aℓ). Only w supplies cycle information independent of amplitude. The restricted design matrix X = [1, c, Iℓ = 2, Iℓ = 3] is nominally full rank for a single global linear cycle trend because w varies within each level; however, that coefficient is identified only through the six within-level contrasts and depends on the imposed linearity. A flexible cycle-history effect and an amplitude effect cannot be uniquely separated by this protocol. The methodological issue is therefore identification, not the size of the Pearson correlation alone. Figure 4 shows the structure.

The consequence is quantitative. The between-level share of sensitivity variance was calculated as ηB = Σℓ nℓ(S^−^ℓ − S^−^)^2^/ΣℓΣk(Sℓ,k − S^−^)^2^, where S^−^ℓ is the mean sensitivity at level ℓ, S^−^ is the record mean, and nℓ = 3. This share is 60% to 92%, with a median near 86%. A linear model of sensitivity against cycle number fitted to such data passes a straight line through a response that is substantially a step function, and its coefficient of determination reflects the between-level step as well as any within-level trend. The high coefficients of determination obtained for the smoothest records are therefore not evidence of an independently identified cycle effect.

After amplitude is held fixed, the estimated within-level slopes are small and inconsistent in sign both within and between specimens, and in one record, the two levels give slopes of opposite sign. The between-level step keeps the same sign in every record. Three cycles per level are insufficient to distinguish a genuinely small cycle effect from a cycle effect that is unresolved by the available repeated observations. The present data therefore identify non-separability under the adopted protocol rather than the absence of a cycle effect. The term apparent cyclic drift is used for the across-cycle change observed under this coupled design.

This is not a claim that earlier studies necessarily misattributed their findings. It is a methodological caution concerning what can and cannot be inferred from a particular design. The present records show that, when a few cycles are applied at ascending amplitude levels, an observed trend with cycle number is compatible with an amplitude response whose share cannot be determined from the same protocol. The comparison studies considered address different material systems or physical problems; they were considered as methodological context but were not used as direct evidence for the present electrical mechanism. Separating the two effects requires a design with independent amplitude and cycle information, and an alternative fit to the same confounded observations cannot generate that information.

Table 5 sets out how the characterization is configured in a sample of this literature. The comparison is deliberately not one of gauge factors, because comparing single coefficients across studies is the practice this study identifies as being of limited reliability: a coefficient obtained at one amplitude is not commensurable with one obtained at another unless the loading range and definition are also reported. What the table compares instead is the protocol, which determines whether a reported cyclic trend can be attributed to cycling. Where the number of amplitude levels is small, the cycles per level are few, and the amplitude order is ascending, the confounding remains possible regardless of how carefully the resulting data are fitted.

### An Experimental Design That Separates the Two Effects

Separating a per-cycle effect from an amplitude effect requires that the two cease to be collinear at the level of the model being estimated. The following is a planning design that provides independent within-level cycle information, controls the main loading-history factors and separates specimen replication from repeated measurements.

- At least four distinct amplitudes should span the service range, including one low enough that a detection floor expressed as a fraction of the stress range does not exclude it;
- About twenty-five cycles should be applied at each fixed amplitude as a precision planning value. For a fixed-amplitude block of *n* cycles, the standard error of a linear cycle slope is approximately SE(βcycle) = sw/[n(n^2^ − 1)/12]^1/2^. Using the largest observed within-level standard deviation, sw = 0.163%/MPa, n = 25 gives SE(βcycle) ≈ 0.0045%/MPa per cycle. With 23 residual degrees of freedom, the two-sided 5% reference threshold is about 0.0094%/MPa per cycle, and a nominal 80% power calculation gives a detectable slope of approximately 0.013%/MPa per cycle under independent-error assumptions. This is a planning calculation, not evidence obtained from the present three-cycle records; serial correlation would reduce the effective information;
- The order in which amplitudes are applied should be randomized or counterbalanced across replicate specimens, and the order of continuous and held protocols should also be randomized. This prevents amplitude and protocol from remaining tied to sequence position and allows load-history effects to be estimated separately;
- A fixed reference amplitude should be re-run periodically between blocks, with dwell location and duration prescribed in advance, so that cumulative history is tracked independently of the amplitude currently applied;
- Independent specimens per amplitude and per conditioning state are required if specimen-level slopes or moisture effects are to be estimated. The present data cannot provide a power-based specimen count because there is only one specimen per conditioning state and therefore no estimate of between-specimen variance. At least three independent specimens per state would be a minimum for estimating that variance; the final number should be determined from a pilot variance and a prespecified effect size. Increasing the number of cycles on one specimen cannot compensate for the absence of specimen replication.

## 6. Monotonic Loading Response of the Same Specimens

The cyclic records cannot support a cycle-denominated prediction. The monotonic records of the same three specimens provide a stress-ratio-denominated within-specimen extrapolation under a selected response family. This comparison demonstrates that the data structure differs between the two protocols, but it does not establish broader predictive validity or isolate material effects from all measurement-history effects.

### 6.1. A Two-Mechanism Response Family

The monotonic response of FCR against stress ratio x = σ/σf was described by the four-parameter family F(x) = Ff{w[(1 − exp(−kx))/(1 − exp(−k))] + (1 − w)[(exp(gx) − 1)/(exp(g) − 1)]}, for 0 ≤ x ≤ 1. The first term is a normalized saturating exponential and the second a normalized growing exponential. Ff is the signed response at failure, w is the weight of the saturating member, k is its exhaustion constant, and g is the growth rate of the second member. Each member equals unity at x = 1, so Ff is the scale parameter. The family was chosen to provide an empirical interpolation between two response shapes suggested by the two-path description of conduction and earlier carbon-fibre models [47,48,49,57]. The assumed linear relation between effective inter-particle gap and stress ratio is empirical. The equation is a response model; its weight is not a measured fraction of conduction pathways.

Fitted mechanism weights differ substantially between conditioning states. The saturated specimen fits at a weight of 0.96 towards the saturating member and the oven-dried specimen at 1.00, while the partially saturated specimen fits at 0.06, almost entirely towards the growth member. The C3 value of 1.00 is a boundary solution of the constrained fit, not evidence that 100% of the current follows one physical pathway; uncertainty based on an interior parameter approximation would not be appropriate at that boundary. The fitted weight therefore cannot be interpreted as a water fraction. Direct verification would require, for example, phase-resolved impedance measurements, pore-solution conductivity or an independent measurement sensitive to inter-particle spacing. No relationship between the measured electrical characteristics and the response scale is fitted or claimed.

### 6.2. Within-Specimen Extrapolation

**Figure 5:**
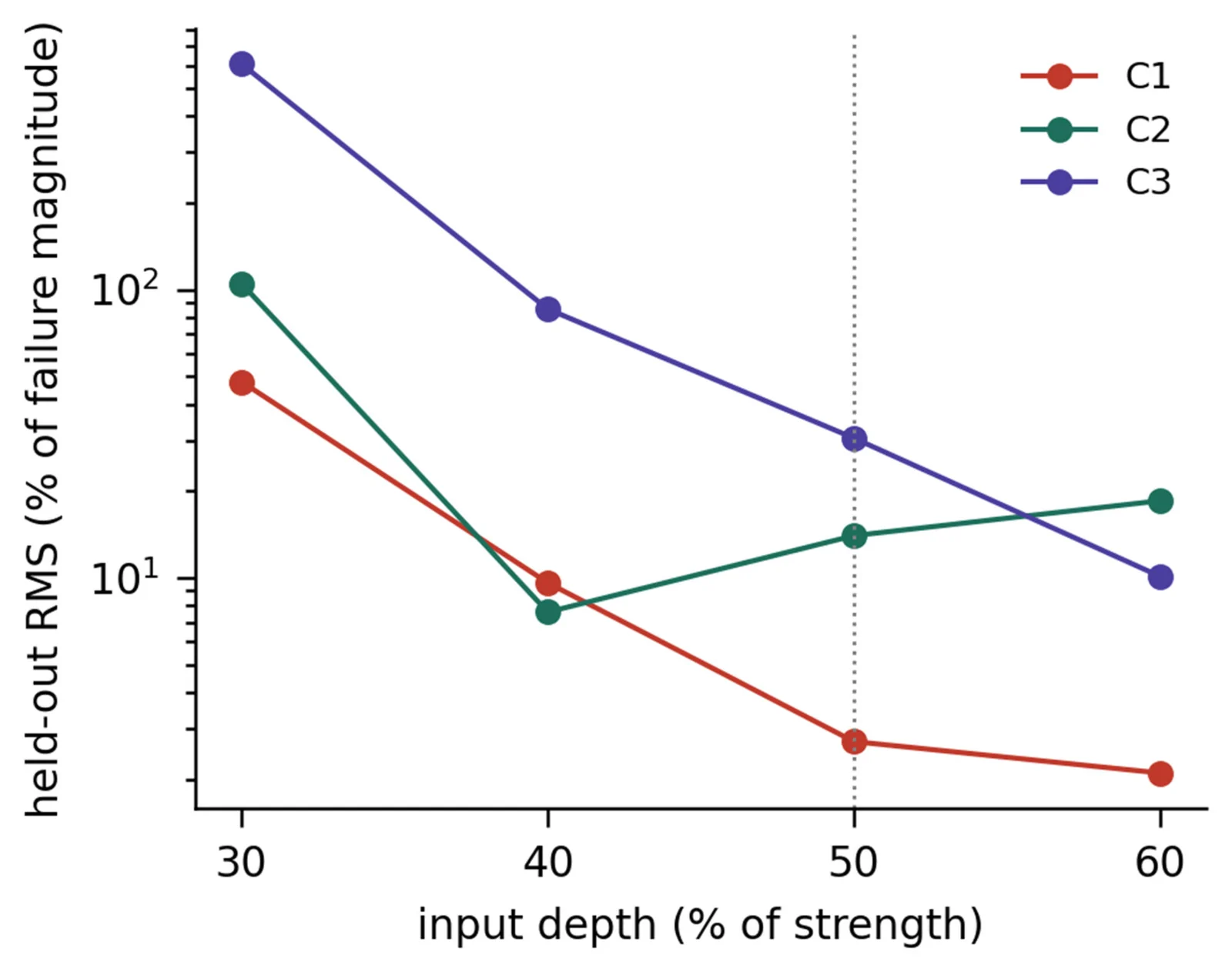
Conditional held-out error against how much of the trajectory was used for the fit, as a percentage of the failure magnitude, on a log scale. The dotted line marks the half-strength cut discussed in the text. Shallow cuts are shown unadjusted; no calibrated prediction interval is implied.

The family was evaluated by truncating each monotonic trajectory at a declared fraction of the specimen strength, fitting on the retained portion only, and comparing the predicted continuation against the held-out remainder. Because the response family was selected after the complete trajectories had been viewed, the held-out portion is not an independent validation set for the model-selection decision. The errors therefore measure conditional within-specimen extrapolation under the selected four-parameter family, not independent predictive validity. Results at four truncation depths are given in Table 6 and plotted in Figure 5. At a truncation depth of half the strength, chosen because the cyclic characterization protocol itself peaks near 49%, the held-out root mean square errors are 2.7%, 14.0%, and 30.5% of the true failure magnitude for C1, C2, and C3, respectively.

Errors at shallower truncation are large and are reported without adjustment: at a depth of 30% the oven-dried specimen has an RMSE of 606.6% of the true failure magnitude and a signed error of +1599.6%. At the half-strength cut the C3 extrapolation overpredicts, with a signed error of +55.4%, so the early portion of the dry trajectory can bias the fitted continuation. No calibrated prediction interval is reported, because the held-out data were not reserved for independent interval calibration; Table 6 reports root mean square error and signed error only.

**Figure 6:**
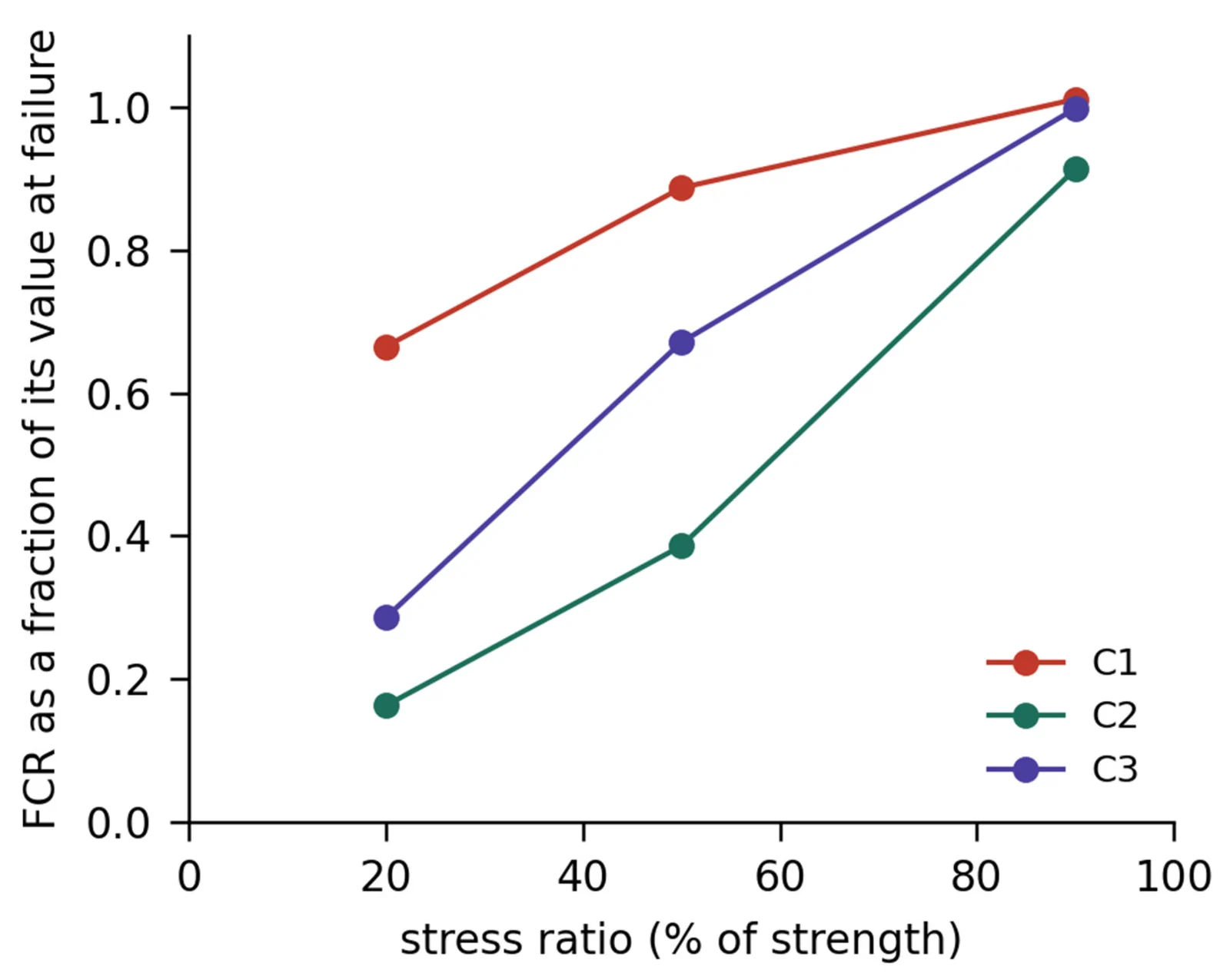
Monotonic response normalized by its value at failure for the three tested specimens. The curves are shown as within-specimen response shapes and are not intended as a general conditioning-state model.

The C2 errors also illustrate why adding measured points does not guarantee better extrapolation under an unspecified or weakly identified response family. Its RMSE decreases from 104.9% at 30% input to 7.6% at 40%, then increases to 14.0% and 18.5% at 50% and 60%. The early C2 trajectory is weak and supplies limited information about the eventual growth rate; adding later points changes the fitted scale and growth parameter to accommodate the observed curvature, which can move the extrapolated endpoint. The non-monotone error sequence is therefore a model-sensitivity result, not evidence of independent predictive reliability. Within these three trajectories, C1, whose response saturates earliest (Figure 6), has the smallest errors at the 50% and 60% cuts. This is a within-specimen observation and should not be generalized to other composites or conditioning histories.

## 7. Discussion

That a loading sensitivity measured on a self-sensing cementitious composite carries with it the amplitude at which it was measured agrees with literature [39,41], and the present per-level uncertainty estimates quantify the differences among amplitude blocks in three individual records. Because amplitude advances with block order under this protocol, those differences are not attributed to amplitude in isolation. This loading sensitivity is distinct from a gauge factor because it is normalized by stress rather than strain. In the C1 record the difference is large: sensitivity magnitude in the lowest-amplitude block tested is more than three times that in the highest. Reporting a single coefficient for such a material without stating the amplitude of measurement omits a variable of the same order as the quantity itself.

What follows from that dependence for the way the coefficient is obtained is the methodological contribution of this paper. The stepped-amplitude protocol makes the between-block part of amplitude and cycle number inseparable by construction. Where a study reports a sensitivity that drifts over cycles and those cycles were applied at ascending amplitude levels, the reported drift is compatible with an amplitude response whose share cannot be determined without additional design information. The present records place the between-level share of sensitivity variance between 60% and 92%, but they do not establish that earlier studies necessarily overstated a cycle effect. A more elaborate fit cannot generate independent variation that the protocol did not provide.

A mechanistic reading is available as a hypothesis and is consistent with some of the monotonic results, although the present data do not test it directly. If conduction in the saturated state proceeds predominantly through the pore solution, load may progressively close that path and reduce the incremental response per unit stress as the path is exhausted. That interpretation is consistent with the saturating member of the response family and with the C1 fitted weight of 0.96, but the fitted weight is not a physical fraction of the current. The partially saturated record shows a weak cyclic amplitude dependence and a fitted weight of 0.06 towards the growing member, but neither observation verifies a tunnelling-dominated pathway. The oven-dried record fits at the boundary w = 1.00, further demonstrating that the model coordinate should not be read as a direct mechanism measurement. Phase-resolved impedance, pore-solution conductivity, and microstructural distance measurements would be needed to test this interpretation.

The direction of the amplitude dependence is not universal, which strengthens the case for reporting it explicitly rather than applying a common correction. The present 0.75% MWCNT concrete records generally show sensitivity magnitude falling as amplitude rises, most steeply in the C1 conditioning history, whereas Roshan et al. [40] reported increasing stress sensitivity with stress level in cement-stabilized sand containing 4% MWCNT/GNP. Because the matrix, filler dosage, electrode arrangement, and conditioning differ, the studies are not direct quantitative comparisons. Each material system should therefore be characterized over its own loading range.

A practical consequence follows for structural health monitoring. Self-sensing elements have been proposed for embedment in beams and for crack self-monitoring in service [36,58,59], and in those applications, the service loading differs from that of a characterization programme. The C1 record provides an experimentally observed example in which a calibration at one amplitude would not transfer to another without appreciable error. Because only one specimen represents each conditioning history, this example is not a general deployment rule. Calibration for these materials should be reported as a response over amplitude rather than as a coefficient, and monitoring systems that assume a fixed gauge factor should state the loading range over which that approximation was checked.

## 8. Limitations

Three specimens were tested, one per conditioning history, with two loading protocols each. There is no replication within a conditioning state, so between-specimen variability cannot be separated from the association with the conditioning history. The nominal temperature and relative humidity histories, conditioning duration, mass-measurement precision, post-conditioning delay and constant-mass status were not retained in the source records. The labels saturated, partially saturated, and oven-dried are therefore operational labels based on the recorded environmental history and mass loss, not direct degree-of-saturation measurements. A larger replicated experiment with controlled conditioning and a common test temperature is required to establish generality.

Three cycles per amplitude level provide limited replication. Under comparison-specific Welch thresholds, it resolves the upper step in three of the six records and the lower step in two, leaving the remaining differences unresolved. The same limited replication constrains any statement about per-cycle behavior at fixed amplitude. The present data therefore support non-identifiability under the coupled protocol rather than absence of a cycle effect. Confidence intervals for the within-level slopes were not reconstructed because the supplied source document retained summary standard deviations rather than the complete branch-level sensitivity values.

The cyclic programme reaches about half of the monotonic strength. No statement is made about the response at higher amplitudes and the fitted responses should not be extrapolated beyond the range tested. The proposed 25-cycle design is a nominal within-specimen precision calculation based on the largest observed within-level standard deviation; it is not a specimen-level power calculation.

The two loading protocols differ in the presence of a sustained hold at both extremes of each cycle, with an achieved dwell that varies between specimens because the hold was terminated manually. The source summary retains median dwells of 18.6, 11.9, and 18.0 s for C1, C2, and C3, but not the complete branch-by-branch dwell distribution. The order of the continuous and held tests was also not retained. Any contrast attributed to the protocol therefore confounds hold location, dwell duration and possible sequence carryover. Future experiments should prescribe and record dwell duration and randomize protocol order.

The monotonic extrapolation results rest on three trajectories, and the response family was selected after inspection of all three complete trajectories. Within-specimen extrapolation does not eliminate that selection bias. The held-out errors are conditional checks under the selected family, not independent predictive validation, and no calibrated prediction interval is claimed. A full validation study requires trajectories not seen during family selection, several candidate response families selected by a prespecified rule, and independent specimens.

The block-to-block variation reported in Section 4 is consistent with an effect already reported and modelled in other systems. Its contribution here is the per-level treatment with explicit within-record uncertainty, the factor-level identification analysis and the reanalysis of a previously reported programme across three conditioning histories. The present paper does not claim a new percolation threshold or a direct physical decomposition of conduction pathways. The methodological claim in Section 5 is the principal new contribution.

## 9. Conclusions

- Under the stepped-amplitude protocol conventional in this field, cycle number and peak stress correlate at r = 0.87 to 0.90, and 60% to 92% of the variance in loading sensitivity lies between amplitude levels rather than within them. The design does not identify a general cycle-history effect independently of amplitude; it does not establish that the cycle effect is zero. Separation requires many repeated cycles at fixed amplitudes, counterbalanced amplitude and protocol order, and independent specimen replication.
- The loading sensitivity of the 0.75% multi-walled carbon nanotube concrete examined here differs among the amplitude blocks of each tested record; because amplitude and block order are not separated by this protocol, the differences are not attributed to amplitude alone. Between the middle and the highest block, sensitivity magnitude falls in all six records, with standardized differences of 2.3 to 11.2; three exceed their comparison-specific Welch critical value. These are within-record findings from three individual specimens.
- At the lower amplitude step, two of the six record-level contrasts exceed their comparison-specific Welch critical value, including +0.673%/MPa at 21.5 standard errors against a critical value of 3.11 for the continuous C1 record. The partially saturated C2 records show no resolvable lower-step change. The differences among C1, C2, and C3 are associations across one specimen per conditioning history and should not be interpreted as a replicated moisture effect.
- At three cycles per level, the Welch degrees of freedom fall between 2.0 and 4.0; on that basis, the lower-step response is resolved for two of the six records and undetermined for the other four. The within-level slopes are insufficient to distinguish a genuinely small cycle effect from an unresolved effect. Three amplitude levels permit a first inspection of response shape, but they do not identify a general amplitude curve or a cycle-history term.
- The direction of amplitude dependence is not universal across the cited literature, so no single correction can be applied. Each material system requires characterization over amplitude with its loading, conditioning, electrode, and measurement conditions stated explicitly.
- Monotonic loading of the same specimens provides a conditional within-specimen extrapolation under the selected four-parameter family, with held-out RMSE values of 2.7%, 14.0%, and 30.5% of the failure magnitude from half-strength input. The 30% C3 input gives an RMSE of 606.6% and a signed error of +1599.6%, while C2 error increases from 7.6% at 40% input to 14.0% and 18.5% at 50% and 60%. These results do not constitute independent predictive validation or a general reliability rule.

## Figures and Tables

**Table 1:** Mix proportions.

Constituent	Quantity	Note
CEM I 52.5R	350 kg/m^3^	EN 197-1:2011
Natural sand	875 kg/m^3^	0 to 4 mm
Coarse aggregate	980 kg/m^3^	4 to 10 mm
Water	175 kg/m^3^	w/c = 0.50 by mass
Superplasticiser	4% by cement mass	polycarboxylic ether
MWCNT	0.75% by cement mass	Nanocyl, Belgium

**Table 2:** Operational conditioning histories and recorded water-mass loss.

State	Condition	Water Mass Loss
C1	20 °C, RH approx. 100%	0%
C2	23 °C, 50% < RH < 100%	2.2%
C3	40 °C, RH < 50%	5.1%

**Table 3:** Loading sensitivity by amplitude level. Values are mean sensitivity in %/MPa with the within-level standard deviation over three cycles in parentheses, at mean peak stress in MPa. The fit slope is that of signed sensitivity against peak stress, so a positive slope denotes a fall in response magnitude as amplitude rises.

Record	Level 1	Level 2	Level 3	Fit Slope, R^2^
C1 continuous	−1.320 (0.026)	−0.647 (0.047)	−0.421 (0.037)	0.0614, 0.956
C1 held	−1.213 (0.163)	−0.731 (0.154)	−0.477 (0.111)	0.0496, 0.855
C2 continuous	−0.406 (0.007)	−0.436 (0.031)	−0.368 (0.002)	0.0020, 0.154
C2 held	−0.378 (0.025)	−0.392 (0.006)	−0.379 (0.006)	−0.0002, 0.005
C3 continuous	−0.722 (0.084)	−0.621 (0.031)	−0.452 (0.031)	0.0179, 0.809
C3 held	−0.475 (0.045)	−0.570 (0.017)	−0.423 (0.015)	0.0025, 0.054

**Table 4:** Adjacent level differences in loading sensitivity, %/MPa, with the standard error of the difference and the ratio of the two. A positive difference denotes a fall in response magnitude as amplitude rises. Each SE ratio is listed with the comparison-specific two-sided 5% Welch critical value t_0.975_,ν and, in parentheses, the Welch–Satterthwaite degrees of freedom ν computed as in Section 4.1. A difference is resolved only where the ratio exceeds its own critical value, which holds for C1 continuous at both steps, C1 held at the lower step, and C3 continuous, and C3 held at the upper step.

Record	Low to Mid	SE Ratio/t_0.975_,ν (ν)	Mid to High	SE Ratio/t_0.975_,ν (ν)
C1 continuous	+0.673 (0.031)	21.5/3.11 (3.1)	+0.226 (0.035)	6.5/2.84 (3.8)
C1 held	+0.482 (0.130)	3.7/2.78 (4.0)	+0.254 (0.110)	2.3/2.89 (3.6)
C2 continuous	−0.029 (0.018)	1.6/3.94 (2.2)	+0.067 (0.018)	3.7/4.27 (2.0)
C2 held	−0.014 (0.015)	1.0/3.90 (2.2)	+0.013 (0.005)	2.7/2.78 (4.0)
C3 continuous	+0.101 (0.052)	1.9/3.54 (2.5)	+0.169 (0.025)	6.8/2.78 (4.0)
C3 held	−0.095 (0.028)	3.5/3.52 (2.6)	+0.147 (0.013)	11.2/2.79 (3.9)

**Table 5:** Characterization protocols reported for self-sensing cementitious composites, compared by experimental configuration rather than by reported sensitivity.

Study	Material System	Amplitude Levels	Cycles per Level	Amplitude Order	Sensitivity Reported as	Confound Present
This work	MWCNT concrete, 0.75 wt%	3	3	ascending	response over amplitude, per level	yes, quantified
[44]	Plain and damaged concrete, Wheatstone bridge	3	3	ascending	single coefficient	yes
[45,46]	MWCNT concrete (source programme)	3	3	ascending	single coefficient	yes
[32]	CNF and hybrid CNF/CF cement	-	-	ascending	single gauge factor	likely
[35]	Steel-fibre industrial smart concrete	-	-	ascending	single strain sensitivity	likely
[56]	Rubber-aggregate concrete	-	-	ascending	single coefficient	likely
[39]	Cement-based, resistance and capacitance	two regimes	-	ascending	amplitude-dependent, 1.7 to 0.4 × 10^−5^ Pa^−1^	dependence reported
[41]	Cement-based sensors	continuous	-	monotonic	non-linear Ramberg–Osgood model	not applicable
[40]	Cement-stabilized sand with carbon nanomaterials	-	-	ascending	increases with stress level	dependence reported, opposite sign

**Table 6:** Within-specimen extrapolation. Held-out root mean square error and signed error in the predicted failure magnitude, both as percentages of the true failure magnitude, at four truncation depths.

Truncation Depth	C1 Saturated	C2 Partially Saturated	C3 Oven-Dried
30%	47.9/+71.5	104.9/+254.8	606.6/+1599.6
40%	9.6/+10.5	7.6/+13.9	85.6/+172.1
50%	2.7/+0.1	14.0/−22.1	30.5/+55.4
60%	2.1/−1.1	18.5/−26.6	10.1/+18.7

## Data Availability

The raw electrical and mechanical records underlying this reanalysis, together with the PiezoMatrix (version 0.1.0, commit a7ad239) and VigilMatrix (commit 372cc7d) analysis tools, are available from the corresponding author on reasonable request. The available request package includes the processed level summaries and the documented analysis definitions for resistance referencing, peak height recovery, amplitude clustering, regression and the calculations used for Table 3, Table 4, Table 5 and Table 6 and Figure 2, Figure 3, Figure 4, Figure 5 and Figure 6. The source programme did not preserve a persistent public repository, the complete branch-level raw values, the numerical prominence setting, the instrument acquisition metadata or the exact software versions and dependencies. These reproducibility limitations are stated explicitly in the manuscript.
